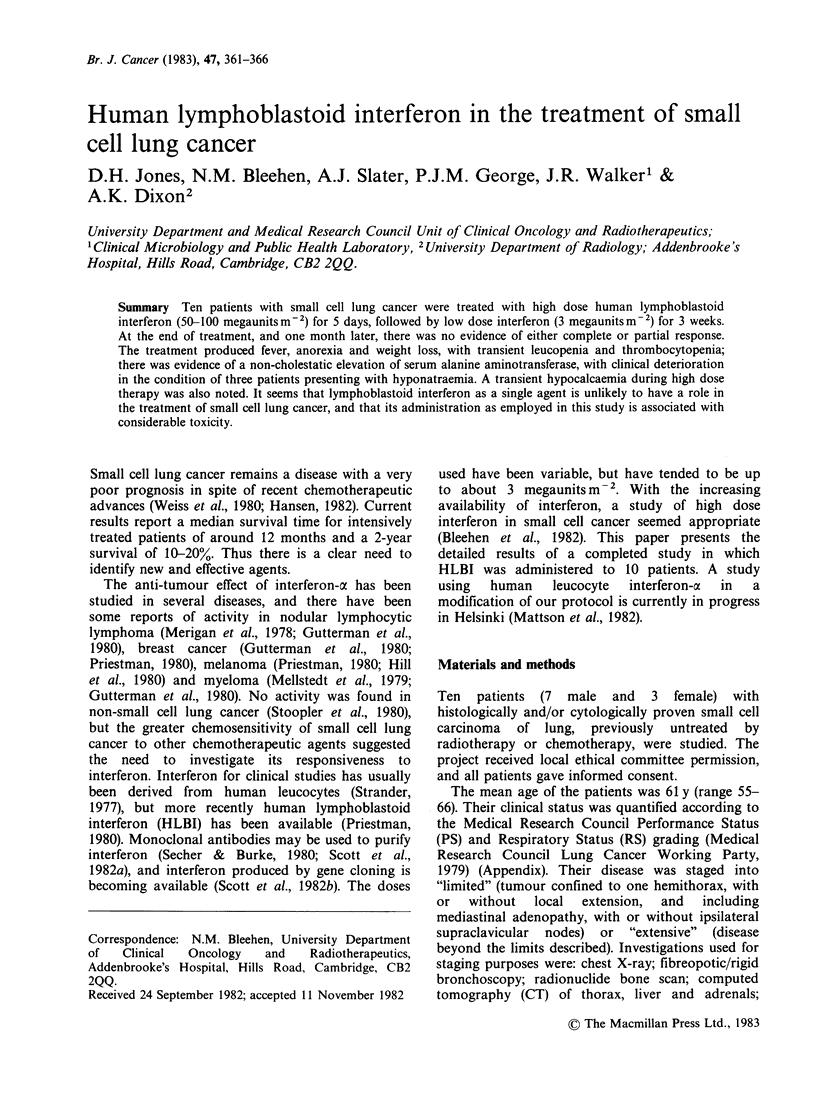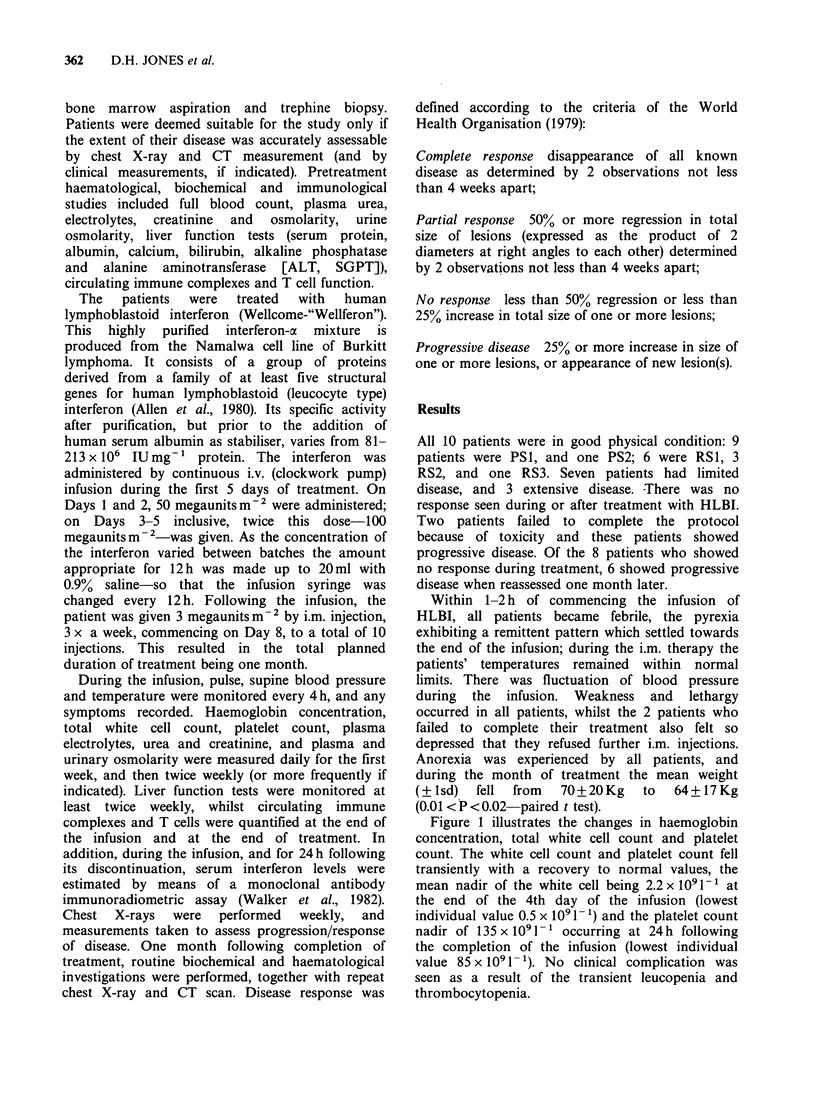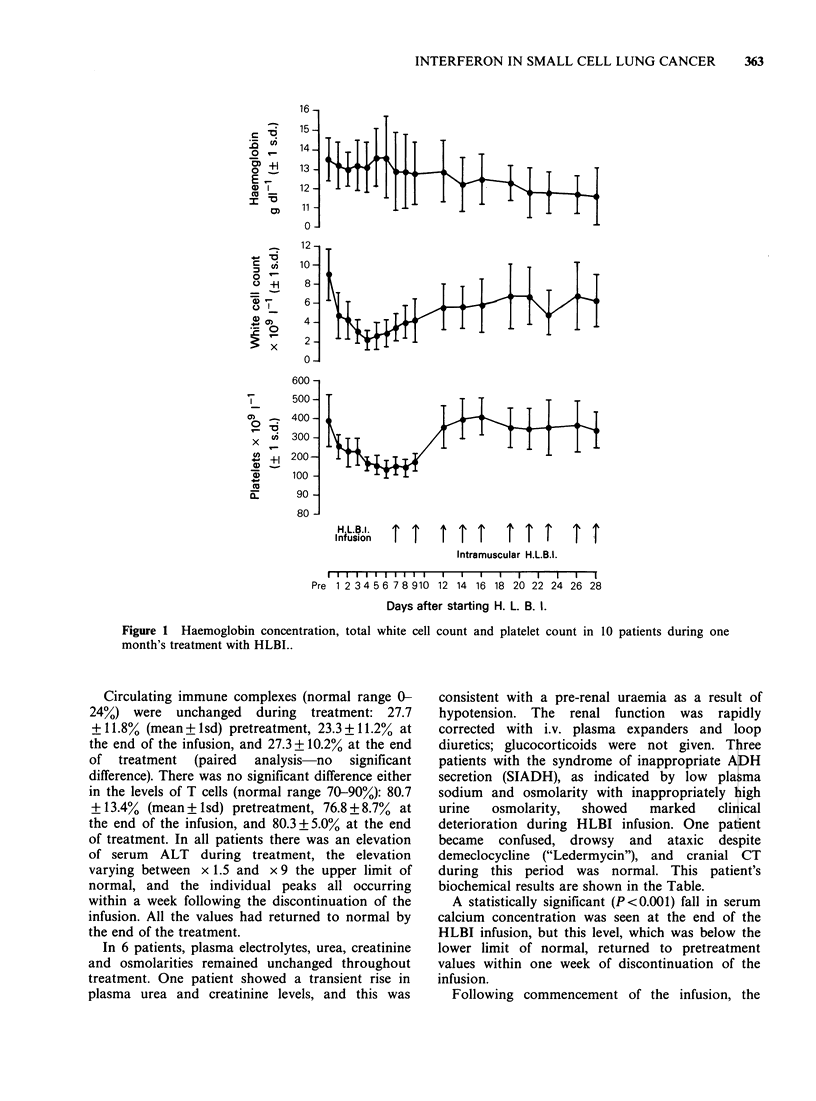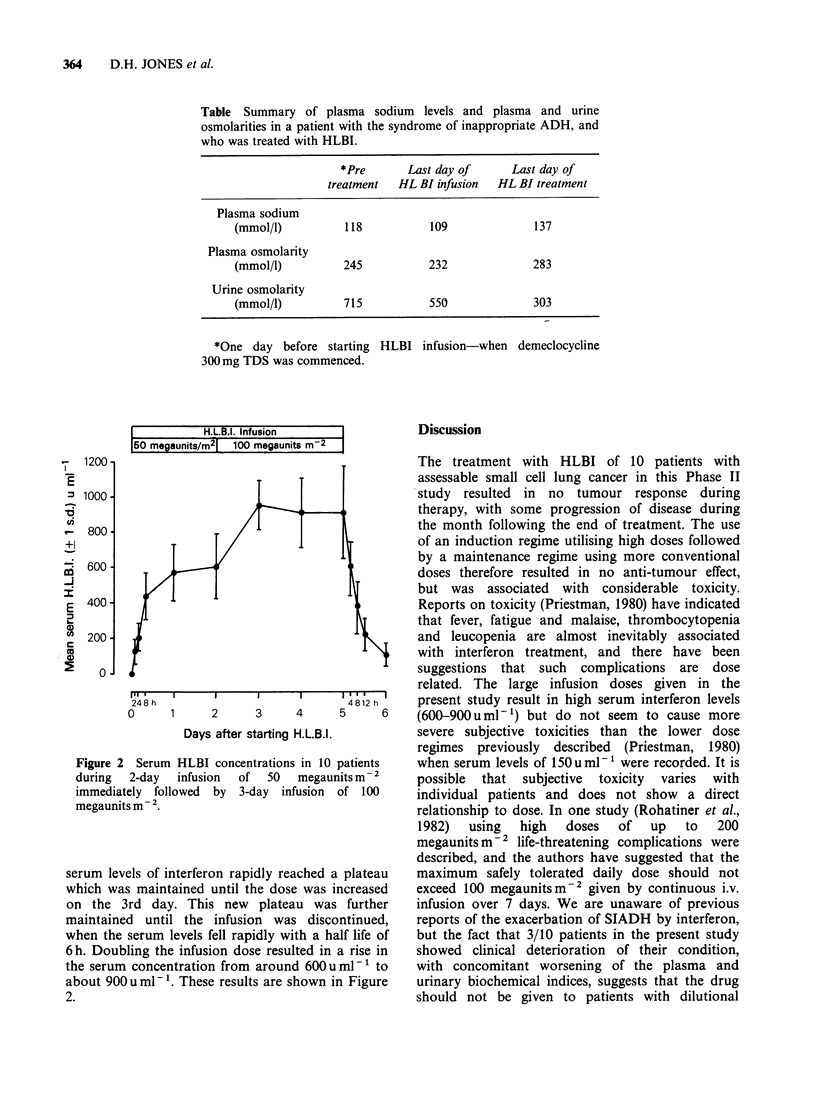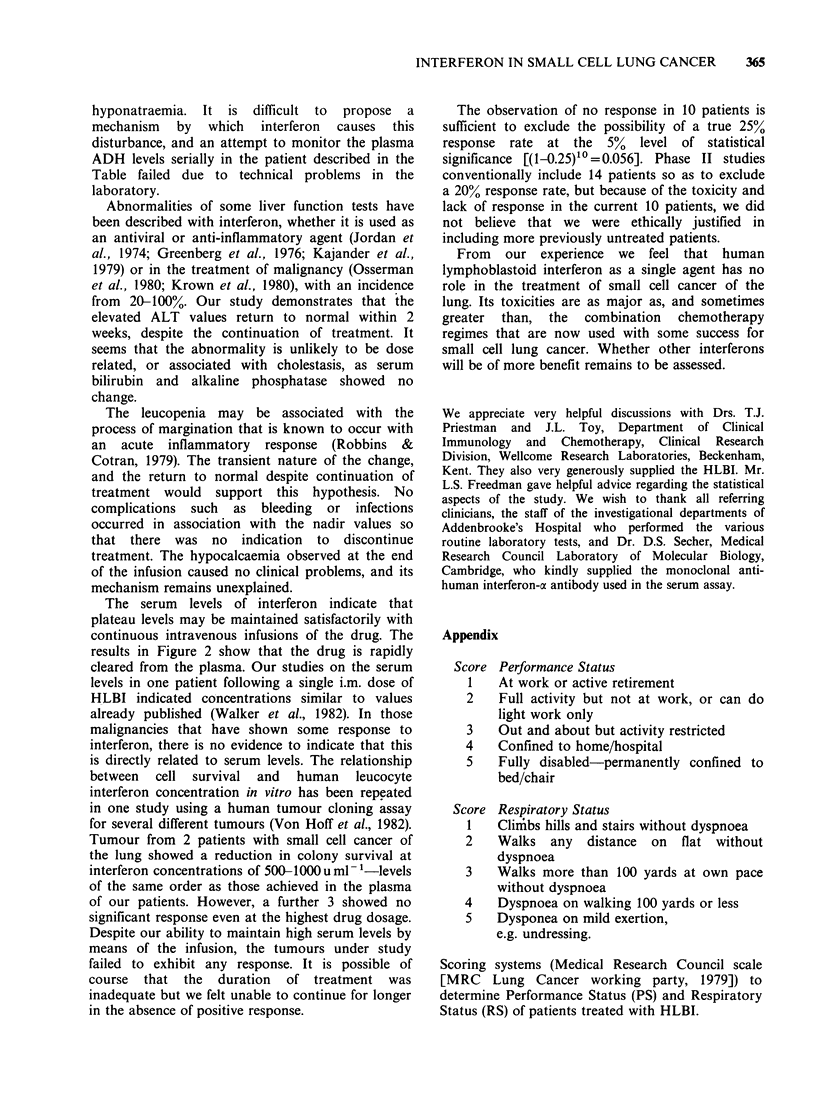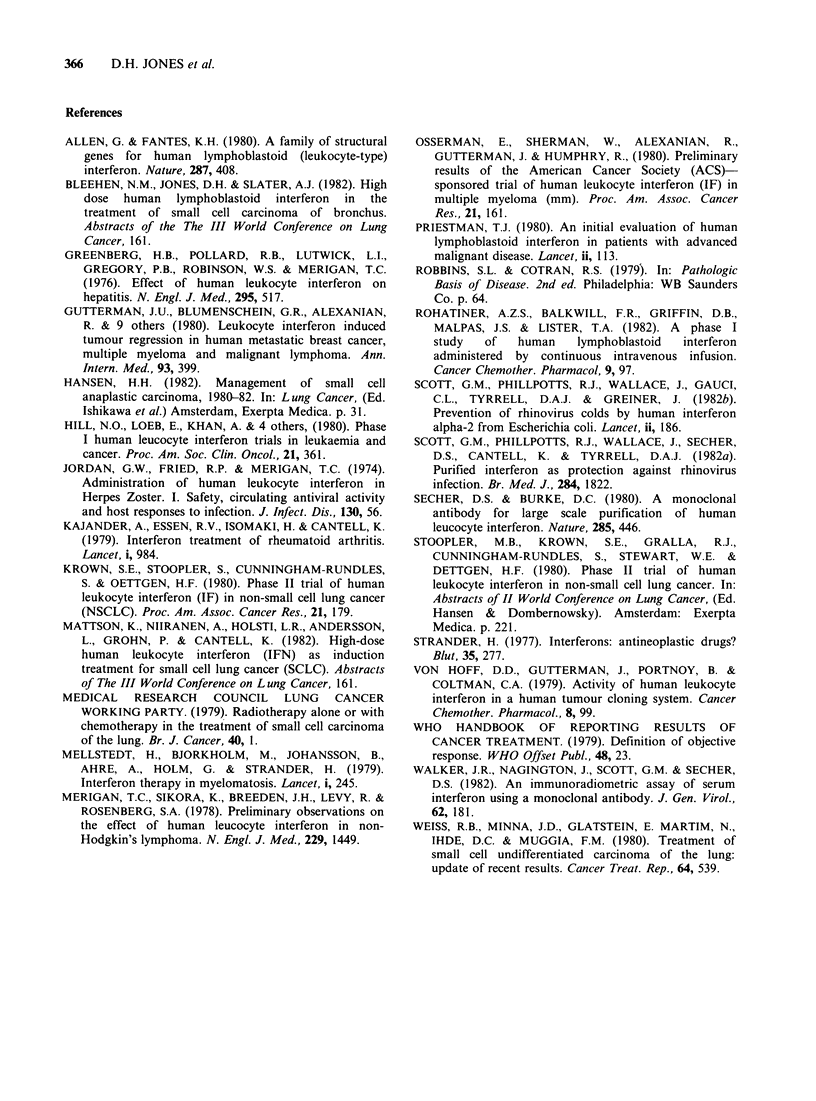# Human lymphoblastoid interferon in the treatment of small cell lung cancer.

**DOI:** 10.1038/bjc.1983.54

**Published:** 1983-03

**Authors:** D. H. Jones, N. M. Bleehen, A. J. Slater, P. J. George, J. R. Walker, A. K. Dixon

## Abstract

Ten patients with small cell lung cancer were treated with high dose human lymphoblastoid interferon (50-100 megaunits m-2) for 5 days, followed by low dose interferon (3 megaunits m-2) for 3 weeks. At the end of treatment, and one month later, there was no evidence of either complete or partial response. The treatment produced fever, anorexia and weight loss, with transient leucopenia and thrombocytopenia; there was evidence of a non-cholestatic elevation of serum alanine aminotransferase, with clinical deterioration in the condition of three patients presenting with hyponatraemia. A transient hypocalcaemia during high dose therapy was also noted. It seems that lymphoblastoid interferon as a single agent is unlikely to have a role in the treatment of small cell lung cancer, and that its administration as employed in this study is associated with considerable toxicity.


*Br. J. Cancer* (1983), **47**, 361–366

### Human lymphoblastoid interferon in the treatment of small cell lung cancer

D.H. Jones, N.M. Bleehen, A.J. Slater, P.J.M. George, J.R. Walker[1] & A.K. Dixon[2]

*University Department and Medical Research Council Unit of Clinical Oncology and Radiotherapeutics; [1]Clinical Microbiology and Public Health Laboratory, [2]University Department of Radiology; Addenbrooke's Hospital, Hills Road, Cambridge, CB2 2QQ.*

**Summary** Ten patients with small cell lung cancer were treated with high dose human lymphoblastoid interferon (50–100 megaunits $m^{-2}$) for 5 days, followed by low dose interferon (3 megaunits $m^{-2}$) for 3 weeks. At the end of treatment, and one month later, there was no evidence of either complete or partial response. The treatment produced fever, anorexia and weight loss, with transient leucopenia and thrombocytopenia; there was evidence of a non-cholestatic elevation of serum alanine aminotransferase, with clinical deterioration in the condition of three patients presenting with hyponatraemia. A transient hypocalcaemia during high dose therapy was also noted. It seems that lymphoblastoid interferon as a single agent is unlikely to have a role in the treatment of small cell lung cancer, and that its administration as employed in this study is associated with considerable toxicity.

Small cell lung cancer remains a disease with a very poor prognosis in spite of recent chemotherapeutic advances (Weiss *et al*., 1980; Hansen, 1982). Current results report a median survival time for intensively treated patients of around 12 months and a 2-year survival of 10–20%. Thus there is a clear need to identify new and effective agents.

The anti-tumour effect of interferon-α has been studied in several diseases, and there have been some reports of activity in nodular lymphocytic lymphoma (Merigan *et al*., 1978; Gutterman *et al*., 1980), breast cancer (Gutterman *et al*., 1980; Priestman, 1980), melanoma (Priestman, 1980; Hill *et al*., 1980) and myeloma (Mellstedt *et al*., 1979; Gutterman *et al*., 1980). No activity was found in non-small cell lung cancer (Stoopler *et al*., 1980), but the greater chemosensitivity of small cell lung cancer to other chemotherapeutic agents suggested the need to investigate its responsiveness to interferon. Interferon for clinical studies has usually been derived from human leucocytes (Strander, 1977), but more recently human lymphoblastoid interferon (HLBI) has been available (Priestman, 1980). Monoclonal antibodies may be used to purify interferon (Secher & Burke, 1980; Scott *et al*., 1982*a*), and interferon produced by gene cloning is becoming available (Scott *et al*., 1982*b*). The doses used have been variable, but have tended to be up to about 3 megaunits $m^{-2}$. With the increasing availability of interferon, a study of high dose interferon in small cell cancer seemed appropriate (Bleehen *et al*., 1982). This paper presents the detailed results of a completed study in which HLBI was administered to 10 patients. A study using human leucocyte interferon-α in a modification of our protocol is currently in progress in Helsinki (Mattson *et al*., 1982).

### Materials and methods

Ten patients (7 male and 3 female) with histologically and/or cytologically proven small cell carcinoma of lung, previously untreated by radiotherapy or chemotherapy, were studied. The project received local ethical committee permission, and all patients gave informed consent.

The mean age of the patients was 61 y (range 55–66). Their clinical status was quantified according to the Medical Research Council Performance Status (PS) and Respiratory Status (RS) grading (Medical Research Council Lung Cancer Working Party, 1979) (Appendix). Their disease was staged into "limited" (tumour confined to one hemithorax, with or without local extension, and including mediastinal adenopathy, with or without ipsilateral supraclavicular nodes) or "extensive" (disease beyond the limits described). Investigations used for staging purposes were: chest X-ray; fibreopotic/rigid bronchoscopy; radionuclide bone scan; computed tomography (CT) of thorax, liver and adrenals;

Correspondence: N.M. Bleehen, University Department of Clinical Oncology and Radiotherapeutics, Addenbrooke's Hospital, Hills Road, Cambridge, CB2 2QQ.
Received 24 September 1982; accepted 11 November 1982

© The Macmillan Press Ltd., 1983

bone marrow aspiration and trephine biopsy. Patients were deemed suitable for the study only if the extent of their disease was accurately assessable by chest X-ray and CT measurement (and by clinical measurements, if indicated). Pretreatment haematological, biochemical and immunological studies included full blood count, plasma urea, electrolytes, creatinine and osmolarity, urine osmolarity, liver function tests (serum protein, albumin, calcium, bilirubin, alkaline phosphatase and alanine aminotransferase [ALT, SGPT]), circulating immune complexes and T cell function.

The patients were treated with human lymphoblastoid interferon (Wellcome-"Wellferon"). This highly purified interferon-α mixture is produced from the Namalwa cell line of Burkitt lymphoma. It consists of a group of proteins derived from a family of at least five structural genes for human lymphoblastoid (leucocyte type) interferon (Allen *et al.*, 1980). Its specific activity after purification, but prior to the addition of human serum albumin as stabiliser, varies from 81–$213 \times 10^6$ IU $mg^{-1}$ protein. The interferon was administered by continuous i.v. (clockwork pump) infusion during the first 5 days of treatment. On Days 1 and 2, 50 megaunits $m^{-2}$ were administered; on Days 3–5 inclusive, twice this dose—100 megaunits $m^{-2}$—was given. As the concentration of the interferon varied between batches the amount appropriate for 12 h was made up to 20 ml with 0.9% saline—so that the infusion syringe was changed every 12 h. Following the infusion, the patient was given 3 megaunits $m^{-2}$ by i.m. injection, 3 × a week, commencing on Day 8, to a total of 10 injections. This resulted in the total planned duration of treatment being one month.

During the infusion, pulse, supine blood pressure and temperature were monitored every 4 h, and any symptoms recorded. Haemoglobin concentration, total white cell count, platelet count, plasma electrolytes, urea and creatinine, and plasma and urinary osmolarity were measured daily for the first week, and then twice weekly (or more frequently if indicated). Liver function tests were monitored at least twice weekly, whilst circulating immune complexes and T cells were quantified at the end of the infusion and at the end of treatment. In addition, during the infusion, and for 24 h following its discontinuation, serum interferon levels were estimated by means of a monoclonal antibody immunoradiometric assay (Walker *et al.*, 1982). Chest X-rays were performed weekly, and measurements taken to assess progression/response of disease. One month following completion of treatment, routine biochemical and haematological investigations were performed, together with repeat chest X-ray and CT scan. Disease response was defined according to the criteria of the World Health Organisation (1979):

*Complete response* disappearance of all known disease as determined by 2 observations not less than 4 weeks apart;

*Partial response* 50% or more regression in total size of lesions (expressed as the product of 2 diameters at right angles to each other) determined by 2 observations not less than 4 weeks apart;

*No response* less than 50% regression or less than 25% increase in total size of one or more lesions;

*Progressive disease* 25% or more increase in size of one or more lesions, or appearance of new lesion(s).

### Results

All 10 patients were in good physical condition: 9 patients were PS1, and one PS2; 6 were RS1, 3 RS2, and one RS3. Seven patients had limited disease, and 3 extensive disease. There was no response seen during or after treatment with HLBI. Two patients failed to complete the protocol because of toxicity and these patients showed progressive disease. Of the 8 patients who showed no response during treatment, 6 showed progressive disease when reassessed one month later.

Within 1–2 h of commencing the infusion of HLBI, all patients became febrile, the pyrexia exhibiting a remittent pattern which settled towards the end of the infusion; during the i.m. therapy the patients' temperatures remained within normal limits. There was fluctuation of blood pressure during the infusion. Weakness and lethargy occurred in all patients, whilst the 2 patients who failed to complete their treatment also felt so depressed that they refused further i.m. injections. Anorexia was experienced by all patients, and during the month of treatment the mean weight (±1sd) fell from 70±20 Kg to 64±17 Kg ($0.01 < P < 0.02$—paired *t* test).

Figure 1 illustrates the changes in haemoglobin concentration, total white cell count and platelet count. The white cell count and platelet count fell transiently with a recovery to normal values, the mean nadir of the white cell being $2.2 \times 10^9 l^{-1}$ at the end of the 4th day of the infusion (lowest individual value $0.5 \times 10^9 l^{-1}$) and the platelet count nadir of $135 \times 10^9 l^{-1}$ occurring at 24 h following the completion of the infusion (lowest individual value $85 \times 10^9 l^{-1}$). No clinical complication was seen as a result of the transient leucopenia and thrombocytopenia.

**Figure 1** Haemoglobin concentration, total white cell count and platelet count in 10 patients during one month's treatment with HLBI..

Circulating immune complexes (normal range 0–24%) were unchanged during treatment: 27.7 ±11.8% (mean±1sd) pretreatment, 23.3±11.2% at the end of the infusion, and 27.3±10.2% at the end of treatment (paired analysis—no significant difference). There was no significant difference either in the levels of T cells (normal range 70–90%): 80.7 ±13.4% (mean±1sd) pretreatment, 76.8±8.7% at the end of the infusion, and 80.3±5.0% at the end of treatment. In all patients there was an elevation of serum ALT during treatment, the elevation varying between ×1.5 and ×9 the upper limit of normal, and the individual peaks all occurring within a week following the discontinuation of the infusion. All the values had returned to normal by the end of the treatment.

In 6 patients, plasma electrolytes, urea, creatinine and osmolarities remained unchanged throughout treatment. One patient showed a transient rise in plasma urea and creatinine levels, and this was consistent with a pre-renal uraemia as a result of hypotension. The renal function was rapidly corrected with i.v. plasma expanders and loop diuretics; glucocorticoids were not given. Three patients with the syndrome of inappropriate ADH secretion (SIADH), as indicated by low plasma sodium and osmolarity with inappropriately high urine osmolarity, showed marked clinical deterioration during HLBI infusion. One patient became confused, drowsy and ataxic despite demeclocycline ("Ledermycin"), and cranial CT during this period was normal. This patient's biochemical results are shown in the Table.

A statistically significant ($P<0.001$) fall in serum calcium concentration was seen at the end of the HLBI infusion, but this level, which was below the lower limit of normal, returned to pretreatment values within one week of discontinuation of the infusion.

Following commencement of the infusion, the

**Table** Summary of plasma sodium levels and plasma and urine osmolarities in a patient with the syndrome of inappropriate ADH, and who was treated with HLBI.

| | **Pre treatment* | *Last day of HL BI infusion* | *Last day of HL BI treatment* |
|---|---|---|---|
| Plasma sodium (mmol/l) | 118 | 109 | 137 |
| Plasma osmolarity (mmol/l) | 245 | 232 | 283 |
| Urine osmolarity (mmol/l) | 715 | 550 | 303 |

*One day before starting HLBI infusion—when demeclocycline 300 mg TDS was commenced.

**Figure 2** Serum HLBI concentrations in 10 patients during 2-day infusion of 50 megaunits m$^{-2}$ immediately followed by 3-day infusion of 100 megaunits m$^{-2}$.

serum levels of interferon rapidly reached a plateau which was maintained until the dose was increased on the 3rd day. This new plateau was further maintained until the infusion was discontinued, when the serum levels fell rapidly with a half life of 6 h. Doubling the infusion dose resulted in a rise in the serum concentration from around 600 u ml$^{-1}$ to about 900 u ml$^{-1}$. These results are shown in Figure 2.

### Discussion

The treatment with HLBI of 10 patients with assessable small cell lung cancer in this Phase II study resulted in no tumour response during therapy, with some progression of disease during the month following the end of treatment. The use of an induction regime utilising high doses followed by a maintenance regime using more conventional doses therefore resulted in no anti-tumour effect, but was associated with considerable toxicity. Reports on toxicity (Priestman, 1980) have indicated that fever, fatigue and malaise, thrombocytopenia and leucopenia are almost inevitably associated with interferon treatment, and there have been suggestions that such complications are dose related. The large infusion doses given in the present study result in high serum interferon levels (600–900 u ml$^{-1}$) but do not seem to cause more severe subjective toxicities than the lower dose regimes previously described (Priestman, 1980) when serum levels of 150 u ml$^{-1}$ were recorded. It is possible that subjective toxicity varies with individual patients and does not show a direct relationship to dose. In one study (Rohatiner *et al.*, 1982) using high doses of up to 200 megaunits m$^{-2}$ life-threatening complications were described, and the authors have suggested that the maximum safely tolerated daily dose should not exceed 100 megaunits m$^{-2}$ given by continuous i.v. infusion over 7 days. We are unaware of previous reports of the exacerbation of SIADH by interferon, but the fact that 3/10 patients in the present study showed clinical deterioration of their condition, with concomitant worsening of the plasma and urinary biochemical indices, suggests that the drug should not be given to patients with dilutional hyponatraemia. It is difficult to propose a mechanism by which interferon causes this disturbance, and an attempt to monitor the plasma ADH levels serially in the patient described in the Table failed due to technical problems in the laboratory.

Abnormalities of some liver function tests have been described with interferon, whether it is used as an antiviral or anti-inflammatory agent (Jordan *et al.*, 1974; Greenberg *et al.*, 1976; Kajander *et al.*, 1979) or in the treatment of malignancy (Osserman *et al.*, 1980; Krown *et al.*, 1980), with an incidence from 20–100%. Our study demonstrates that the elevated ALT values return to normal within 2 weeks, despite the continuation of treatment. It seems that the abnormality is unlikely to be dose related, or associated with cholestasis, as serum bilirubin and alkaline phosphatase showed no change.

The leucopenia may be associated with the process of margination that is known to occur with an acute inflammatory response (Robbins & Cotran, 1979). The transient nature of the change, and the return to normal despite continuation of treatment would support this hypothesis. No complications such as bleeding or infections occurred in association with the nadir values so that there was no indication to discontinue treatment. The hypocalcaemia observed at the end of the infusion caused no clinical problems, and its mechanism remains unexplained.

The serum levels of interferon indicate that plateau levels may be maintained satisfactorily with continuous intravenous infusions of the drug. The results in Figure 2 show that the drug is rapidly cleared from the plasma. Our studies on the serum levels in one patient following a single i.m. dose of HLBI indicated concentrations similar to values already published (Walker *et al.*, 1982). In those malignancies that have shown some response to interferon, there is no evidence to indicate that this is directly related to serum levels. The relationship between cell survival and human leucocyte interferon concentration *in vitro* has been repeated in one study using a human tumour cloning assay for several different tumours (Von Hoff *et al.*, 1982). Tumour from 2 patients with small cell cancer of the lung showed a reduction in colony survival at interferon concentrations of 500–1000 u $ml^{-1}$—levels of the same order as those achieved in the plasma of our patients. However, a further 3 showed no significant response even at the highest drug dosage. Despite our ability to maintain high serum levels by means of the infusion, the tumours under study failed to exhibit any response. It is possible of course that the duration of treatment was inadequate but we felt unable to continue for longer in the absence of positive response.

The observation of no response in 10 patients is sufficient to exclude the possibility of a true 25% response rate at the 5% level of statistical significance [$(1-0.25)^{10}=0.056$]. Phase II studies conventionally include 14 patients so as to exclude a 20% response rate, but because of the toxicity and lack of response in the current 10 patients, we did not believe that we were ethically justified in including more previously untreated patients.

From our experience we feel that human lymphoblastoid interferon as a single agent has no role in the treatment of small cell cancer of the lung. Its toxicities are as major as, and sometimes greater than, the combination chemotherapy regimes that are now used with some success for small cell lung cancer. Whether other interferons will be of more benefit remains to be assessed.

We appreciate very helpful discussions with Drs. T.J. Priestman and J.L. Toy, Department of Clinical Immunology and Chemotherapy, Clinical Research Division, Wellcome Research Laboratories, Beckenham, Kent. They also very generously supplied the HLBI. Mr. L.S. Freedman gave helpful advice regarding the statistical aspects of the study. We wish to thank all referring clinicians, the staff of the investigational departments of Addenbrooke's Hospital who performed the various routine laboratory tests, and Dr. D.S. Secher, Medical Research Council Laboratory of Molecular Biology, Cambridge, who kindly supplied the monoclonal anti-human interferon-α antibody used in the serum assay.

### Appendix

| *Score* | *Performance Status* |
|---|---|
| 1 | At work or active retirement |
| 2 | Full activity but not at work, or can do light work only |
| 3 | Out and about but activity restricted |
| 4 | Confined to home/hospital |
| 5 | Fully disabled—permanently confined to bed/chair |

| *Score* | *Respiratory Status* |
|---|---|
| 1 | Climbs hills and stairs without dyspnoea |
| 2 | Walks any distance on flat without dyspnoea |
| 3 | Walks more than 100 yards at own pace without dyspnoea |
| 4 | Dyspnoea on walking 100 yards or less |
| 5 | Dysponea on mild exertion, e.g. undressing. |

Scoring systems (Medical Research Council scale [MRC Lung Cancer working party, 1979]) to determine Performance Status (PS) and Respiratory Status (RS) of patients treated with HLBI.

### References

ALLEN, G. & FANTES, K.H. (1980). A family of structural genes for human lymphoblastoid (leukocyte-type) interferon. *Nature*, **287**, 408.

BLEEHEN, N.M., JONES, D.H. & SLATER, A.J. (1982). High dose human lymphoblastoid interferon in the treatment of small cell carcinoma of bronchus. *Abstracts of the The III World Conference on Lung Cancer*, 161.

GREENBERG, H.B., POLLARD, R.B., LUTWICK, L.I., GREGORY, P.B., ROBINSON, W.S. & MERIGAN, T.C. (1976). Effect of human leukocyte interferon on hepatitis. *N. Engl. J. Med.*, **295**, 517.

GUTTERMAN, J.U., BLUMENSCHEIN, G.R., ALEXANIAN, R. & 9 others (1980). Leukocyte interferon induced tumour regression in human metastatic breast cancer, multiple myeloma and malignant lymphoma. *Ann. Intern. Med.*, **93**, 399.

HANSEN, H.H. (1982). Management of small cell anaplastic carcinoma, 1980–82. In: *Lung Cancer*, (Ed. Ishikawa *et al.*) Amsterdam, Exerpta Medica. p. 31.

HILL, N.O., LOEB, E., KHAN, A. & 4 others, (1980). Phase I human leucocyte interferon trials in leukaemia and cancer. *Proc. Am. Soc. Clin. Oncol.*, **21**, 361.

JORDAN, G.W., FRIED, R.P. & MERIGAN, T.C. (1974). Administration of human leukocyte interferon in Herpes Zoster. I. Safety, circulating antiviral activity and host responses to infection. *J. Infect. Dis.*, **130**, 56.

KAJANDER, A., ESSEN, R.V., ISOMAKI, H. & CANTELL, K. (1979). Interferon treatment of rheumatoid arthritis. *Lancet*, **i**, 984.

KROWN, S.E., STOOPLER, S., CUNNINGHAM-RUNDLES, S. & OETTGEN, H.F. (1980). Phase II trial of human leukocyte interferon (IF) in non-small cell lung cancer (NSCLC). *Proc. Am. Assoc. Cancer Res.*, **21**, 179.

MATTSON, K., NIIRANEN, A., HOLSTI, L.R., ANDERSSON, L., GROHN, P. & CANTELL, K. (1982). High-dose human leukocyte interferon (IFN) as induction treatment for small cell lung cancer (SCLC). *Abstracts of The III World Conference on Lung Cancer*, 161.

MEDICAL RESEARCH COUNCIL LUNG CANCER WORKING PARTY. (1979). Radiotherapy alone or with chemotherapy in the treatment of small cell carcinoma of the lung. *Br. J. Cancer*, **40**, 1.

MELLSTEDT, H., BJORKHOLM, M., JOHANSSON, B., AHRE, A., HOLM, G. & STRANDER, H. (1979). Interferon therapy in myelomatosis. *Lancet*, **i**, 245.

MERIGAN, T.C., SIKORA, K., BREEDEN, J.H., LEVY, R. & ROSENBERG, S.A. (1978). Preliminary observations on the effect of human leucocyte interferon in non-Hodgkin's lymphoma. *N. Engl. J. Med.*, **229**, 1449.

OSSERMAN, E., SHERMAN, W., ALEXANIAN, R., GUTTERMAN, J. & HUMPHRY, R., (1980). Preliminary results of the American Cancer Society (ACS)—sponsored trial of human leukocyte interferon (IF) in multiple myeloma (mm). *Proc. Am. Assoc. Cancer Res.*, **21**, 161.

PRIESTMAN, T.J. (1980). An initial evaluation of human lymphoblastoid interferon in patients with advanced malignant disease. *Lancet*, **ii**, 113.

ROBBINS, S.L. & COTRAN, R.S. (1979). In: *Pathologic Basis of Disease. 2nd ed.* Philadelphia: WB Saunders Co. p. 64.

ROHATINER, A.Z.S., BALKWILL, F.R., GRIFFIN, D.B., MALPAS, J.S. & LISTER, T.A. (1982). A phase I study of human lymphoblastoid interferon administered by continuous intravenous infusion. *Cancer Chemother. Pharmacol*, **9**, 97.

SCOTT, G.M., PHILLPOTTS, R.J., WALLACE, J., GAUCI, C.L., TYRRELL, D.A.J. & GREINER, J. (1982*b*). Prevention of rhinovirus colds by human interferon alpha-2 from Escherichia coli. *Lancet*, **ii**, 186.

SCOTT, G.M., PHILLPOTTS, R.J., WALLACE, J., SECHER, D.S., CANTELL, K. & TYRRELL, D.A.J. (1982*a*). Purified interferon as protection against rhinovirus infection. *Br. Med. J.*, **284**, 1822.

SECHER, D.S. & BURKE, D.C. (1980). A monoclonal antibody for large scale purification of human leucocyte interferon. *Nature*, **285**, 446.

STOOPLER, M.B., KROWN, S.E., GRALLA, R.J., CUNNINGHAM-RUNDLES, S., STEWART, W.E. & DETTGEN, H.F. (1980). Phase II trial of human leukocyte interferon in non-small cell lung cancer. In: *Abstracts of II World Conference on Lung Cancer*, (Ed. Hansen & Dombernowsky). Amsterdam: Exerpta Medica. p. 221.

STRANDER, H. (1977). Interferons: antineoplastic drugs? *Blut*, **35**, 277.

VON HOFF, D.D., GUTTERMAN, J., PORTNOY, B. & COLTMAN, C.A. (1979). Activity of human leukocyte interferon in a human tumour cloning system. *Cancer Chemother. Pharmacol.*, **8**, 99.

WHO HANDBOOK OF REPORTING RESULTS OF CANCER TREATMENT. (1979). Definition of objective response. *WHO Offset Publ.*, **48**, 23.

WALKER, J.R., NAGINGTON, J., SCOTT, G.M. & SECHER, D.S. (1982). An immunoradiometric assay of serum interferon using a monoclonal antibody. *J. Gen. Virol.*, **62**, 181.

WEISS, R.B., MINNA, J.D., GLATSTEIN, E. MARTIM, N., IHDE, D.C. & MUGGIA, F.M. (1980). Treatment of small cell undifferentiated carcinoma of the lung: update of recent results. *Cancer Treat. Rep.*, **64**, 539.